# Characterization of *Triticum turgidum* sspp. *durum*, *turanicum*, and *polonicum* grown in Central Italy in relation to technological and nutritional aspects

**DOI:** 10.3389/fpls.2023.1269212

**Published:** 2023-12-06

**Authors:** Samuela Palombieri, Marco Bonarrigo, Alessandro Cammerata, Giulia Quagliata, Stefania Astolfi, Domenico Lafiandra, Francesco Sestili, Stefania Masci

**Affiliations:** ^1^ Department of Agriculture and Forest Science (DAFNE), University of Tuscia, Viterbo, Italy; ^2^ Council for Agricultural Research and Economics, Research Centre for Engineering and Agro-Food Processing, Rome, Italy

**Keywords:** *Triticum turgidum*, *Triticum durum*, *Triticum turanicum*, *Triticum polonicum*, technological quality, micronutrients, fiber, root traits

## Abstract

**Introduction:**

Wheat is a staple food, with the two most common species being *Triticum aestivum* and *Triticum turgidum* ssp*. durum*. Moreover, the latter, *T. turgidum*, includes other tetraploid subspecies, among which the sspp. *turanicum* (Khorasan wheat) and *polonicum* (Polish wheat), whose importance has increased in the last decades, representing alternative crops for marginal areas, in addition to being a source of genetic diversity.

**Methods:**

In this work, different accessions of these three subspecies of *T. turgidum* have been grown in 2 years in the same environment and have been characterized for technological properties and factors affecting nutritional quality, such as fiber amount and the content of micro- and macro-nutrients in grains, and for root morphological traits.

**Results:**

These analyses allowed the identification, in particular, of a Polish wheat accession showing better technological performances, a higher amount of positive micro- and macro-elements, and a lower amount of toxic cadmium. The modern variety Svevo and the Polish Pol2 showed the lowest and the highest shoot:root ratio, respectively. The high shoot:root ratio in Pol2 was mainly attributable to the decrease in root growth. Although Pol2 had a lower root biomass, its particular root morphology made it more efficient for nutrient uptake, as evident from the greater accumulation of micro- and macro-nutrients.

**Discussion:**

These results underline that it is not possible to draw general conclusions about the difference between primitive and modern wheats, but rather a case-by-case approach should be chosen.

## 1 Introduction

Wheat is one of the oldest cultivated plants and one of the most important sources of food. Under this name, different species are included, two of which are the most commonly grown: *Triticum aestivum*, also known as bread wheat, because mostly used for bread and leavened products, and *Triticum turgidum* ssp*. durum* (also known as *T. durum* or durum wheat), mostly used for pasta or typical Mediterranean foods such as bulghur, couscous, and, in some cases, flat and leavened bread as well.

The genome composition of *T. aestivum* is AABBDD, namely, hexaploidy, whereas that of the tetraploid *T. durum* is AABB. Moreover, *T. durum*, other subspecies of *T. turgidum*, share the same genomic composition, among which there are *T. turgidum* ssp. *polonicum* and *T. turgidum* ssp. *turanicum*, also known, respectively, as *T. polonicum* (or Polish wheat) and *T. turanicum* (or Khorasan wheat, from the Iranian Region where it was first described). They are free-threshing with tough rachis that are easy-harvest wheats, because they have soft glumes and non-hulled seeds (Yang et al., 2022). *T. turanicum* and *T. polonicum* are closely related, and it has been suggested that the first originated from the hybridization with *T. dicoccon* (Michalcová et al., 2014).

The importance of the two subspecies has increased in the last decades, as a consequence of the introduction in the market of KAMUT^®^ brand wheat, an ancient variety of grain (*Triticum turgidum ssp. turanicum*) that has reached a high popularity because of its suggested positive effects on human health (Bordoni et al., 2017; Spisni et al., 2020). In the wake of KAMUT^®^ brand wheat, any Khorasan wheat is acquiring importance because it is considered healthy (Geisslitz and Scherf, 2021), with some accessions sold under registered names and others simply described as such, sold as semolina or processed foods, with a wide market, especially in Western Countries where they can be found even in the large-scale distribution.

Furthermore, both Polish and Khorasan wheats, being able to be grown organically and adaptable to different environments, represent attractive and alternative crops for the marginal areas of Mediterranean basin, which were no longer widely cultivated due to the low grain yields compared with modern wheat cultivars. However, traditional (mostly local) farmers have continued to grow them to use in low-input agriculture, and they are a source of genetic diversity (Ganugi et al., 2021). Moreover, they are important for economic reasons, because heritage wheats respond to the demand of a specific niche of consumers, careful to the need of conserving cultural habits, often associated by people with the concept of a healthier lifestyle.

Heritage wheats are typically consumed as whole wheats, and this contributes to their healthiness. In general, they have poor technological properties. This is due to the poor-quality gluten proteins and to the presence of fibers, mostly present in the outer pericarp. Because heritage wheats are mostly consumed as whole-wheat leavened products, this affects gluten formation and gas-retention capacity, because they absorb most of the water available (Li et al., 2014; Cappelli et al., 2019; Cappelli and Cini, 2021).

Regarding health-related compounds, the situation is rather controversial and strongly dependent on the specific genotype taken into consideration (Shewry and Hey, 2015; Prandi et al., 2017; Ozuna and Barro, 2018; Lovegrove et al., 2020; Shewry et al., 2020), rather than to their belonging to a specific historical period. There is a strong need for further research on this matter, along with a standardization of evaluation procedures, in order to make reliable comparisons (Shewry, 2018).

Khorasan wheat is by far the most known, but Polish wheat has similar characteristics. It is highly adaptable, especially to Mediterranean climates, and, despite the name, it is mostly grown in Andalusia (Rodríguez-Quijano et al., 2003), Italy, Western Ukraine, Southern Asia, Algeria, and Ethiopia (Eticha et al., 2006). It is characterized by a high content of microelements (Bieńkowska et al., 2019) and by a higher amylose content (Rodríguez-Quijano et al., 2003) that accounts for a higher amount of resistant starch that increases the nutritional quality of derived foods.

The aim of this work is the characterization of accessions of Khorasan and Polish wheats, in comparison to durum wheats, grown for 2 years in the same environment, in terms of technological properties and factors affecting nutritional quality, such as fiber amount and the content of micro- and macro-nutrients in grains. This latter aspect will be also related to the root morphological traits of the different genotypes.

## 2 Materials and methods

### 2.1 Plant materials

A previous analysis of 50 accessions of Khorasan and Polish wheats (Sestili et al., 2018) allowed to select five accessions of *T. turanicum* and five accessions of *T. polonicum*, which showed higher quality characteristics. They are listed in **Table 1**, along with the durum wheat cultivars used as references.

**Table 1:** Species, cultivars, and accessions used in this paper.

Species	Original accession code or cultivar	New accession code
*T. turanicum*	PI624217	Tur5
*T. turanicum*	PI184543	Tur21
*T. turanicum*	PI576854	Tur26
*T. turanicum*	Kamut^®^	Khor1*
*T. turanicum*	Etrusco	Etrusco
*T. polonicum*	PI191810	Pol2
*T. polonicum*	PI191808	Pol6
*T. polonicum*	CLTR5023	Pol11
*T. polonicum*	AS304	Pol304
*T. polonicum*	IC12196	Pol2156
*T. durum*	Aureo	–
*T. durum*	Svevo	–
*T. durum*	Senatore Cappelli	–

*Khor1 corresponds to kernels obtained by local field cultivation of KAMUT^®^ brand wheat, purchased by Molino Bongiovanni s.r.l (Villanova Mondovì, CN, Italy). KAMUT^®^ trademark is used to market and sell the QK-77 variety with certain quality guarantees. Because KAMUT^®^ is a registered trademark of Kamut International Ltd. and Kamut Enterprises of Europe bv, this name could not be used for locally produced kernels because they were obtained out of the official KAMUT^®^ food chain.

Selected materials were grown in 2 years (2018–2019 and 2019–2020) at the experimental educational farm “Nello Lupori” at the University of Tuscia, in three randomized blocks (1.5 m × 2 m), and pools from each block correspond to the biological replicates. Not all the accessions were used for all the analyses, and they are specified in the text.

Whole flour for the gluten protein and nutritional related analyses was obtained by using a Cyclotec mill (Foss Tecator AB, Hoganas, Sweden) equipped with a 0.5-mm sieve, whereas, in the case of technological analyses, a traditional roller milling process (Bühler, model MLU 202, Uzwil, Switzerland) was used. Each grain sample was conditioned by adding water until the moisture value of 17% and left to rest for 24 h. The main milling products were collected: semolina refined through a sieving treatment (sieve types: 38GG, 40GG, and 44GG) using a suitable pilot plant sieving system (NAMAD Impianti, Rome, Italy), bran fractions (coarse and refined types), and fine middling.

### 2.2 Technological analyses

Grain samples were evaluated for test weight, moisture and protein content, gluten percentage, and yellow color through Near-Infrared analysis in Transmission mode by using Infratec™ mod 1241 (FOSS, Hillerød, Denmark), by using less than 60 s for 10 subsamples, including specific gravity analysis and maximum of 40 s for subsamples, with dynamic subsampling technology.

The evaluation of the yellow color was performed with the reflection colorimeter Konica Minolta Chroma Meter CR-400 with an attachment CR-A50 for the measurements of granular material (space L *, a *, and b *); the color was expressed by yellow index. The average was calculated on the 10 different subsamples. Gluten quantity and quality were evaluated by Gluten Index direct method using Glutomatic System (Perten, Sweden) (ICC 158). The rheological parameters were evaluated by using the Chopin alveograph (model NG) AACC Method 54-30.02 (1999) by performing four replicates. For pasta-making process, a pilot press plant (NAMAD Impianti, Rome, Italy) was used, and the “spaghetti” format (Ø =1.6 mm) has been adopted. The end-products (pasta samples) were submitted to a drying cycle for 18 h at low temperature (50°C) using a suitable dryer plan (AFREM, Lyon, France). Pasta evaluation was carried out using the method modified by D'Egidio et al. (1993). Statistical analyses [analysis of variance (ANOVA) and Tukey test] were performed by using the software PAST (v. 2.12, Oslo, Norway) (Hammer et al., 2001).

### 2.3 Mineral content

The amounts of zinc (Zn), magnesium (Mg), iron (Fe), copper (Cu), cadmium (Cd), and selenium (Se) were determined by the acid digestion method followed by detection with Inductively Coupled Plasma Optical Emission 184 Spectrometer (ICP-OES). In detail, for each sample, three technical replicates of whole flour were subjected to a first phase of acid digestion using a high-pressure laboratory microwave oven (Mars plus CEM, Italy) with a 1,800-W energy output. A pre-digestion was carried out on 200 mg of dry sample in three consecutive steps: 1 h with 7.5 mL of HNO_3_ at 67% v/v, 1 h with 0.5 mL of HCl; 30 min with 2 mL of H_2_O_2_ to reach a final volume of 10 mL. The acid solution obtained was mineralized within the microwave, increasing the temperature from 25°C to 180°C in 37 min and maintaining the final temperature for 15 min. After cooling, each sample was diluted in a volume of 25 mL with high-purity water (18 MΩ/cm) and then filtered with a 0.45-mm–pore size filter. Trace elements’ quantifications were performed using an ICP-OES with an axial configuration (8,000 DV, Perkin Elmer) equipped with an ultrasonic nebulizer (Teledyne Cetac Technologies, Omaha, USA). Calibration standards were prepared and processed in the same way as samples up to the dilution step and were used to evaluate the concentration levels of trace elements (multi-element standard solution, CaPurAn, CPAchem, Stara Zagora, Bulgaria). The frequencies used for the determinations were as follows: Zn, 213.9 nm; Mg, 279.0 nm; Fe, 259.9 nm; Cu, 324.7 nm; Cd, 228.8 nm; and Se, 196 nm, through a specific accuracy analyzed with the use of ERM-BC21 (the European reference material).

### 2.4 Arabinoxylan content

The total (TOT-AX) and the water-extractable arabinoxylans (WE-AX) were measured on whole flours using the colorimetric methods reported respectively by Douglas (1981) and by Finnie et al. (2006) with some modification according to Romano et al. (2022). For each biological sample relative to each genotype, three technical replicates were done; TOT-AXs and WE-AXs were expressed as percentage of xylose using the following equations:


$$TOTAXs=\left(\frac{\text{mg}}{\text{g}}\right)=\frac{\text{absorbance }\left(552-510\right)-\text{q}}{m}*200$$

$$WEAXs=\left(\frac{\text{mg}}{\text{g}}\right)=\frac{\text{absorbance }\left(552-510\right)-\text{q}}{m}*200$$

where m is the slope and q is the intercept of the xylose standard curve.

### 2.5 β-Glucan

The β-glucan content was determined on whole flour using the β-Glucan Assay Kit (Mixed Linkage) (K-BGLU, Megazyme Ireland Ltd.). For each genotype, three technical replicates were analyzed.

### 2.6 Statistical analysis

Two different two-way ANOVA analyses (α = 0.05) were used: one to assess the effect of genotype and year along with their interaction, and one to assess the effect of subspecies and year and their interaction as well. Mineral element data were log-transformed to ensure ANOVA assumptions. Significant differences were evaluated using Tukey’s HSD (honestly significant difference) test (α = 0.05). To evaluate the contribution of every variable to the total variance, the percentage of their sum of squares on the total was calculated.

Statistical analyses and plots of contents of mineral elements, arabinoxylans, and β-glucan were made with R using the packages *Rmisc*, *Agricolae*, and *ggplot2* (Wickham, 2016; Mendiburu, 2021; Hope, 2022; R Core Team, 2022).

### 2.7 Characterization of different genotypes at juvenile stage

#### 2.7.1 Plant growing conditions

Seeds of the different genotypes were allowed to germinate in aeroponics in the dark at 28°C for 4 days. Uniform seedlings were selected and transferred in hydroponic culture in plastic pots (six seedlings per pot), containing 2 L of a nutrient solution (Quagliata et al., 2023). Plants were grown under controlled conditions with a day/night cycle of 16 h/8 h at 28°C/20°C air temperature, 80% relative humidity, and 200 µE m^−2^ s^−1^ light intensity. After 5 days from sowing, plants were harvested and analyzed.

#### 2.7.2 Determination of chlorophyll level

At harvest, chlorophyll level was determined on the youngest fully expanded leaf using a non-destructive portable apparatus, the Soil Plant Analysis Development (SPAD-502 Plus, Konica Minolta, Osaka, Japan). The values are provided as SPAD units.

#### 2.7.3 Analysis of root morphological traits

Five days after sowing, the root apparatus of each genotype was excised from the stem and analyzed using the software WinRHIZO (Regent Instruments Inc., Quebec, Canada) and an Epson scanner as described by Quagliata et al. (2023).

#### 2.7.4 Visualization of iron deposits in roots using the Perls stain

The localization of ferric iron (Fe^3+^) in root tissues of the different genotypes was performed as described by Frittelli et al. (2023). Briefly, excised roots were incubated with a 2% K_4_[Fe(CN)_6_] and 2% HCl solution for 15 min at room temperature. Localization of Fe^3+^ was observed with a Zeiss (Oberkochen, Germany) Axioscop II microscope equipped with anepifluorescence illuminator (HBO50/AC BP 390-420 exciter filter and LP 450 barrier filter).

#### 2.7.5 Statistical analysis relative to roots measurements

The results were presented as mean ± standard deviation (SD) of three independent biological replicates (n = 3). All data were statistically analyzed by one-way ANOVA with Student’s t-test at p< 0.05 by using the statistical software CoStat (version 6.45).

The hierarchical clustering was performed on the datasets by complete method and Euclidean distance measurement in R (R Core Team, 2022).

## 3 Results

### 3.1 Technological characterization of the tetraploid genotypes

**Figure 1:**
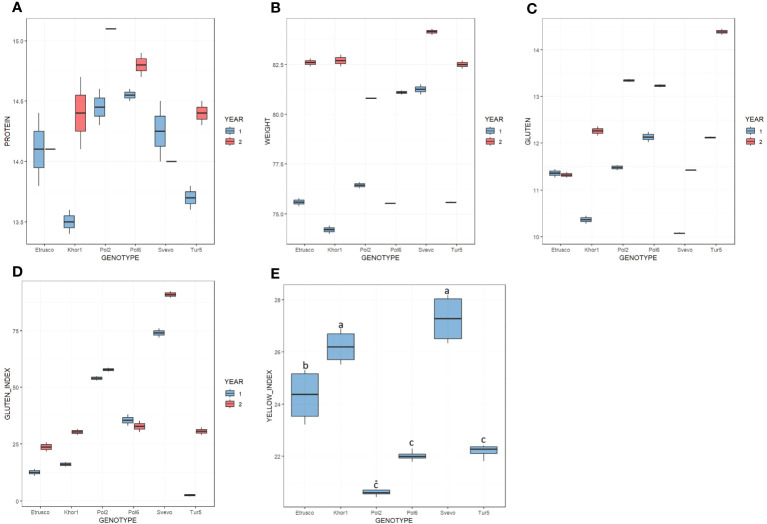
Boxplot of quality parameters on six genotypes in the 2 years (first year in light blue; second year in red). **(A)** Protein content % d.m. **(B)** Test weight kg/hL. **(C)** Gluten % d.m. **(D)** Gluten Index. **(E)** Yellow Index. In this latter case, a single color has been used to indicate that there is no significative difference among the 2 years but only among genotypes.

The qualitative parameters summarized in **Figure 1** and **Supplementary Table 1** were evaluated in two growing seasons. In the first year, test weight values ranged from 74.2 kg/hL to 81.3 kg/hL, whereas, in the second year, from 80.8 kg/hL to 84.2 kg/hL. In both seasons, Svevo was the genotype with the highest value of test weight, whereas no significant difference was observed among the accessions of *T. turgidum* ssp. *turanicum* and *polonicum*. In the 2 years, the protein content varied between 13.5% and 15.1% dry matter. ANOVA analysis did not highlight significant differences between the two growing seasons and between the different genotypes in this regard. The gluten amount varied in the range from 10.1% to 14.4% and, in general, was higher in the second year except for Etrusco, which had similar values in the two growing seasons. Moreover, the Gluten Index was influenced by the growing season and showed a higher value in the second year for all accessions, except for Pol6 that showed a slight decrease. In detail, during the 2018–2019 season, the Gluten Index range varied from 3% in Tur5 and reached 74% in Svevo, whereas, during 2019–2020, it varied from 24% in Etrusco up to 91% in Svevo. Among all accessions analyzed, only Tur5 presented a strong difference in Gluten Index between the two growing seasons analyzed. The ANOVA showed for all qualitative parameters, except for the yellow index, a statistically significant influence (*p<* 0.05) by both genotype and year under study. Tukey’s *post-hoc* test was performed on both variables (genotype × year). In particular, for the yellow index, ANOVA analysis highlighted significant differences among accessions but not for growing season (**Figure 1E**). Svevo and Khor1 presented the highest value, whereas Pol2, Pol6, and Tur5 presented the lowest.

### 3.2 Rheological properties of semolina

The alveographic P/L values obtained from semolina of the tetraploid accessions (expect Khor1) were lower compared with that of Svevo in both growing seasons due to the reduced tenacity (P) and increased extensibility (L) of dough (**Table 2**). The values of Svevo and Khor1, on the other hand, showed a reduced extensibility at the expense of a better P/L value in the 2 years (1.66 and 1.12, respectively). In detail, the P/L ratio is lower in the second growing season and ranged from a minimum of 0.32 (Tur5 in the second year) to a maximum of 2.63 (Svevo in the first year). The W parameter showed more homogeneous values during the two crop years from a minimum of 91 (Etrusco, second year) to a maximum of 246 (Svevo, second year) (**Table 2**).

**Table 2:** Rheological parameters obtained from semolina analyzed with Chopin’s alveograph of accessions of *Triticum* subspecies.

Accession code	P		L		W(J·10^-4^)		P/L	
	2018–2019	2019–2020	2018–2019	2019–2020	2018–2019	2019–2020	2018–2019	2019–2020
Etrusco	60	50	65	71	117	91	0.92	0.7
Pol2	57	52	78	95	134	145	0.73	0.55
Pol6	52	42	80	65	127	86	0.65	0.65
Tur5	46	43	107	133	110	124	0.43	0.32
Khor1	n.a.	64	n.a.	57	n.a.	114	n.a.	1.12
Svevo	129	108	49	65	239	246	2.63	1.66

Data relative to Khor1 are available for the second year only. n.a., not available.

### 3.3 Pasta evaluation

Spaghetti, the most common pasta shape worldwide, was evaluated for important quality parameters for consumers such as size, cooking time, and cooked texture (**Table 3**). The spaghetto diameter, which influenced the cooking time of the pasta, showed an increasing value in the second year with Svevo and Khor1 having the highest values. In the first year, the results showed no differences in the optimal cooking times among genotypes. In the second year, however, this parameter showed a higher value for Svevo and Tur5 and a general decrease compared with that in the previous season. The looseness had differences among accessions in both years with lower values during the second season. Etrusco and Svevo showed the highest value (80) in the first year, followed by Pol2 and Tur5 (75). Differently, Tur5 and Khor1 had the best looseness (70) in the second season, followed by Svevo and Etrusco (60). The bulkiness showed a decrease during the second years with Khor1 presenting the highest value (55), whereas the other accessions showed the same value (50). In general, the parameters used for pasta evaluation were better in the first year than in the second for all the genotypes.

**Table 3:** Pasta values of three subsepcies of *Triticum turgidum*.

Accession code	Spaghetto diameter (mm)	Optimal cooking times		Stickiness		Firmness		Bulkiness	
		2018–2019	2019–2020	2018–2019	2019–2020	2018–2019	2019–2020	2018–2019	2019–2020
Etrusco	1.52–1.58	9′40″	6′45″	80	70	80	60	75	50
Pol2	1.55–1.68	9′30″	6′40″	65	50	75	55	65	50
Pol6	1.54–1.58	9′35″	6′30″	70	50	70	50	75	50
Tur5	1.50–1.60	9′30″	7′30″	70	50	75	70	70	50
Khor1	1.65–1.72	n.d.	6′15″	n.d.	75	n.d.	70	n.d.	55
Svevo	1.62–1.66	9′40″	8′45″	70	50	80	60	70	50

Data relative to Khor1 are available for the second year only. n.d., not determined.

### 3.4 Characterization of mineral content in tetraploid genotypes

**Figure 2:**
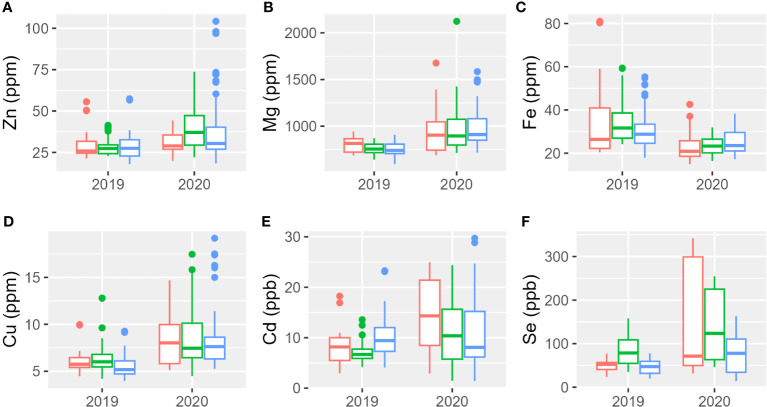
Boxplot of mineral elements content of the three subspecies studied in 2 years. In red, *T. durum*; in green, *T. polonicum*; and in blue, *T. turanicum*. **(A)** Zinc; **(B)** magnesium; **(C)** iron; **(D)** copper; **(E)** cadmium; **(F)** selenium.

The analysis of the micro-elements (Zn, Fe, Cu, Cd, and Se) and macro-elements (Mg) was performed using the acid digestion method coupled with the ICP-OES technique on the different tetraploid accessions listed in **Table 1**; Svevo, Aureo, and Senatore Cappelli were used as reference genotypes. Results analyses are reported in **Figure 2**, **Supplementary Figures 1**, **2**, and **Supplementary Tables 2**, **3**. The mineral content was higher during the second year in which showed a higher range of variability among genotypes. Only iron showed an opposite trend: it presented higher concentrations either in the genotypes or greater variability in the first year than in the second. **Table 4** reports the percentages of the sum of squares of each variable of the two ANOVA models. The first ANOVA analysis, which considered single accessions, highlighted significant effects on the content of each mineral by genotype, year, and interaction between them. Se was the only element whose variance was mainly explained by genotype. The variances of Zn and Cd were mainly explained by the interaction between genotype and year. Lastly, the variances of Mg, Fe, and Cd were mainly explained by year. The second ANOVA analysis (**Table 4**, **Supplementary Figure 1**), which considered the different subspecies *T. durum*, *T. turanicum*, and *T. polonicum*, highlighted significant differences on the content of Cd and Se in three subgroups. The first was significantly higher in durum and Khorasan wheats than that in the Polish wheats, whereas the second was significantly higher in *T. polonicum* and significantly lower in *T. turanicum* than that in the others. There were no significant differences among subspecies for the other minerals. Zn, Fe, and Cd showed significant effects of the interaction between genotypes and year. For all the minerals, the effect of the year among subspecies was significant. Taking into consideration individual accessions, during the first growing season, the only mineral whose concentration did not show changes among genotypes was Zn; whereas, during the second year, all minerals showed a different accumulation among genotypes (**Supplementary Table 3**). During 2018–2019, Khor1 had the lowest accumulation of Zn, whereas Tur5 had the highest accumulation. The highest accumulation of Mg occurred during the second growing season where Senatore Cappelli showed a lower accumulation in this mineral. Higher values were found in Pol2156>Khor1>Aureo>Tur21>Svevo. In the first year, the lowest accumulation of iron was found in Tur21, whereas the highest accumulation was found in Senatore Cappelli>Pol2>Pol304. In the second year, on the contrary, Senatore Cappelli showed the second lowest Fe accumulation, whereas Aureo>Tur26>Tur5 exhibited the highest. Senatore Cappelli is also characterized by a high accumulation of Cd and a low accumulation of Cu. Cd showed a high accumulation in Tur5, a low accumulation in Pol2. Finally, in both growing seasons, the accumulation of Se was lowest in Tur5 and highest in Pol304.

**Table 4:** Minerals content of the three subsepcies of *Triticum turgidum*.

Variable	Transformation	Sums of Squares 1				Sums of Squares 2			
		Genotype	Year	Genotype × Year	Residual	Subspecies	Year	Subspecies × Year	Residual
Zn	log	23.92***	12.88***	24.10***	39.09	1.45^ns^	12.88***	2.29*	83.36
Mg	log	11.19***	30.52***	16.63***	41.64	0.12^ns^	30.52***	1.15^ns^	68.19
Fe	log	12.23***	22.72***	10.8***	54.23	0.28^ns^	22.72***	2.14*	74.85
Cu	log	19.9***	27.52***	13.94***	38.61	1.13^ns^	27.52***	0.89^ns^	70.44
Cd	log	34.98***	3.22***	35.52***	26.26	2.86**	3.22**	3.65**	90.25
Se	log	58.6***	12.06***	21.70***	7.63	14.79***	12.06***	0.73^ns^	72.40

Percentage of the sum of squares of each variable and of the residuals for the micro- and macro-elements content. Model 1 takes into consideration genotype, whereas model 2 takes into consideration subspecies. *p< 0.05, **p< 0.01, ***p< 0.001, and ^ns^p > 0.05.

### 3.5 Characterization of Arabinoxylan and β-glucan content in tetraploid genotypes

**Figure 3:**
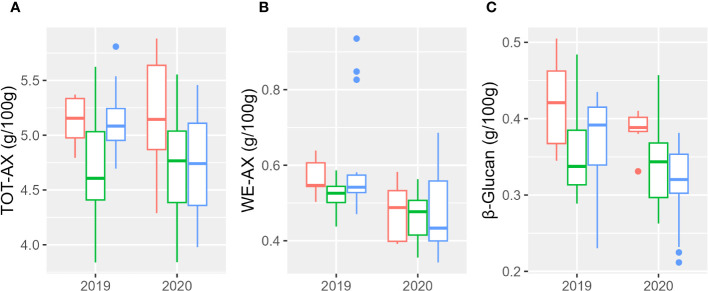
Boxplot of arabinoxylan and β-glucan contents of the three subspecies studied in the 2 years. In red, *T. durum*; in green, *T. polonicum*; and in blue, *T. turanicum.* **(A)** Total arabinoxylan; **(B)** water-extractable arabinoxylan; **(C)** β-glucan.

Results relative to the evaluation of total (TOT-AX) and extractable (WE-AX) arabinoxylans are shown in **Figure 3** and **Supplementary Table 4**. Total arabinoxylan content ranged from 3.84% (w/w) to 5.88% (w/w); the water-extractable content ranged from 0.34% (w/w) to 0.93% (w/w); the β-glucan content ranged from 0.21% (w/w) to 0.50% (w/w). Both β-glucan and WE-AX contents were significantly higher in 2019. **Table 5** reports the percentages of the sum of squares of each variable of the two ANOVA models. Considering the single accessions, the main contribution to the differences in WE-AX and β-glucan content was due to genotype, whereas TOT-AX content was mostly influenced by genotype-by-year interaction, in addition to by genotype. In both growing seasons, Tur26 showed the highest WE-AX content, whereas Senatore Cappelli and Pol304 were among the genotypes that accumulated the highest amount of TOT-AX and β-glucan, respectively (**Supplementary Table 3**). Differently, considering the second ANOVA model, only for TOT-AX and β-glucan, there were significant effects for the subspecies (**Supplementary Figure 3**), and, only for WE-AX and β-glucan, there were significant effects for the year (**Supplementary Figure 4**). No significant effects were found for the interaction subspecies × year. In both growing seasons, durum wheats showed the highest content of β-glucan and TOT-AX, whereas there were no significant difference among the subspecies for the WE-AX content.

**Table 5:** Fiber content of the three subsepcies of *Triticum turgidum*.

Variable	Sums of Squares 1				Sums of Squares 2			
	Genotype	Year	Genotype × Year	Residual	Subspecies	Year	Subspecies × Year	Residual
WE-AX	66.89***	18.31***	10.42***	4.35	5.15^ns^	18.31***	1.55^ns^	74.97
TOT-AX	38.85***	2.25***	30.31***	28.57	12.33**	2.25^ns^	4.70^ns^	80.70
β-Glucan	74.70***	7.17***	7.99***	10.11	15.75**	7.17*	1.62^ns^	75.43

Percentage of the sum of squares of every variable and of the residuals for the fiber content. Model 1 (Sum of Squares 1) takes into consideration genotype, whereas model 2 (Sum of Squares 2) takes into consideration subspecies. *p< 0.05, **p< 0.01, ***p< 0.001, and ^ns^p > 0.05.

### 3.6 Characterization of different genotypes at juvenile stage

#### 3.6.1 Plant growth rate and chlorophyll content

**Figure 4:**
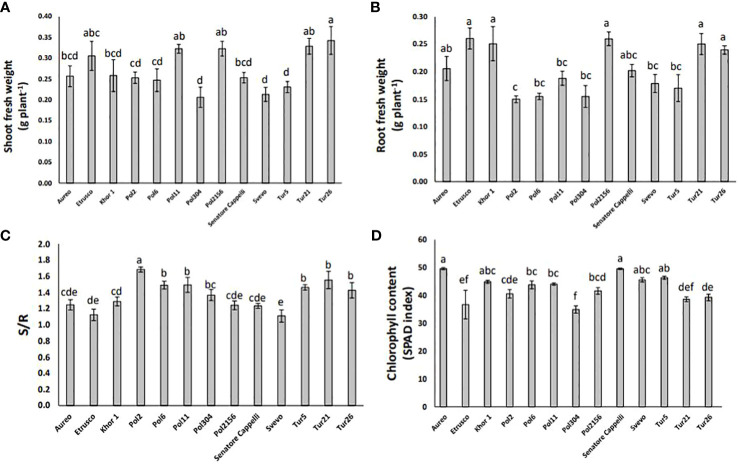
Shoot fresh weight **(A)**, root fresh weight **(B)**, shoot:root ratio **(C)**, and chlorophyll content **(D)** of thirteen genotypes of tetraploid wheat grown hydroponically. Data as reported as mean ± standard deviation (SD) of measurements carried out in triplicate and obtained from three independent experiments (biological replicates). All data were statistically analyzed by one-way analysis of variance (ANOVA) with Student’s t-test (p < 0.05).

The plant growth rate was measured as fresh biomass production of both shoots and roots. The genotypes Pol304, Svevo, and Tur5 showed the lowest shoot biomass production, whereas the genotype Tur26 showed the highest one (**Figure 4A**). In particular, in the genotypes Pol304, Svevo, and Tur5, shoot biomass production was reduced by 40%, 38%, and 33%, respectively, as compared with that in the genotype Tur26 (**Figure 4A**). On the other hand, the genotype Pol2 showed the lowest root fresh biomass production (**Figure 4B**). The shoot-to-root ratio (S/R) plays a crucial role in a plant’s overall growth and development, being directly linked to how a plant allocates its resources, such as nutrients, water, and carbohydrates. The genotypes Svevo and Pol2 showed the lowest and the highest S/R, respectively (**Figure 4C**). Finally, measuring chlorophyll level in leaves of all different genotypes, there was no large variation in SPAD units across this population. In particular, the genotype Pol2156 reached the lowest value, which was 16% lower with respect to Aureo and Senatore Cappelli, the genotypes showing the highest chlorophyll level (**Figure 4D**).

#### 3.6.2 Root morphological traits

**Figure 5:**
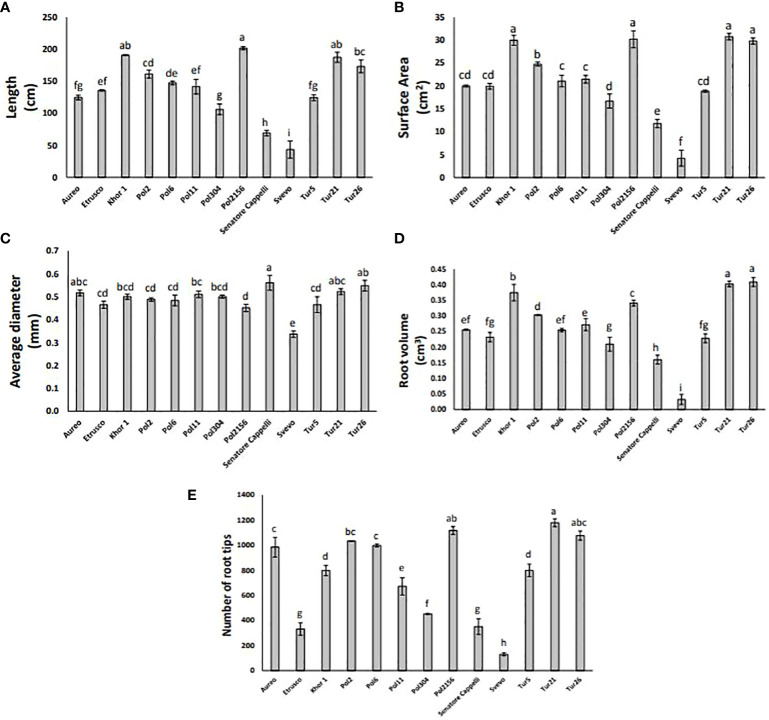
Root length **(A)**, root surface area **(B)**, root average diameter **(C)**, root volume **(D)**, and number of root tips **(E)** of the thirteen tetraploid wheat genotypes at 9 days after sowing. Data as reported as mean ± standard deviation (SD) of measurements carried out in triplicate and obtained from three independent experiments (biological replicates). All data were statistically analyzed by one-way analysis of variance (ANOVA) with Student’s t-test (p< 0.05).

**Figure 6:**
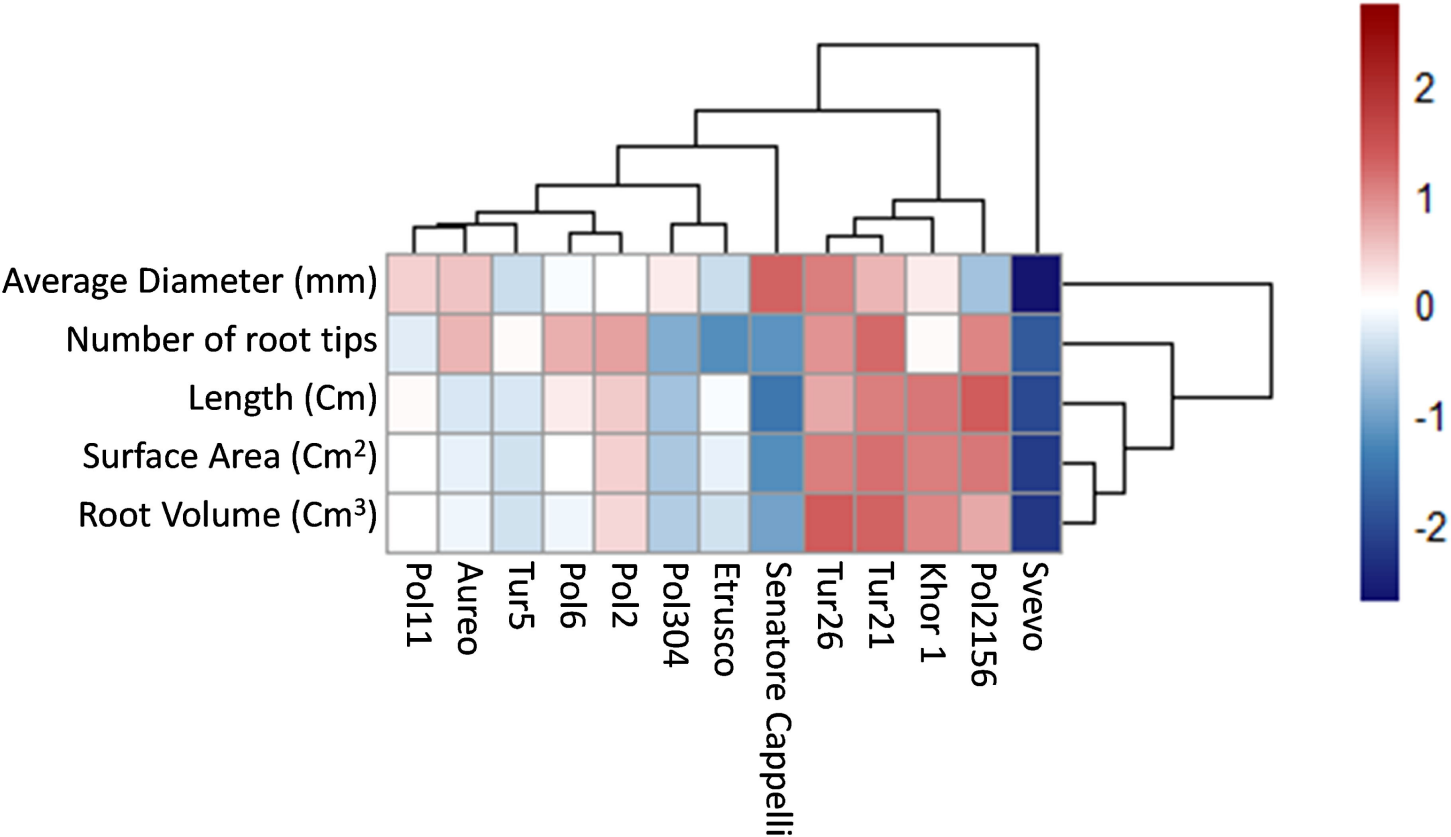
Heatmap representing hierarchical clustering of root traits dataset of thirteen tetraploid wheat genotypes by complete method and Euclidean distance measure. The color bar depicts the gradient of values of roots traits.

Root system architecture is crucial for exploring soil and acquiring resources, as well as for crop adaptation to stress and productivity. Using WinRHIZO scanning equipment, a set of morphological traits was assessed, including length, volume, diameter, surface area, and number of tips of the root systems (**Figure 5**). To facilitate data discussion and identify major clusters across all different genotypes, hierarchical clustering was performed on the morphological traits dataset by complete method and Euclidean distance measurement (**Figure 6**). Results showed that both Tur21 e Tur26 clustered together and showed the highest values of all morphological traits (**Figure 6**). On the other hand, in Svevo, all morphological traits reached the lowest values, although Svevo showed the most balanced S/R (**Figure 6**).

#### 3.6.3 Visualization of iron deposits in root tissues

**Figure 7:**
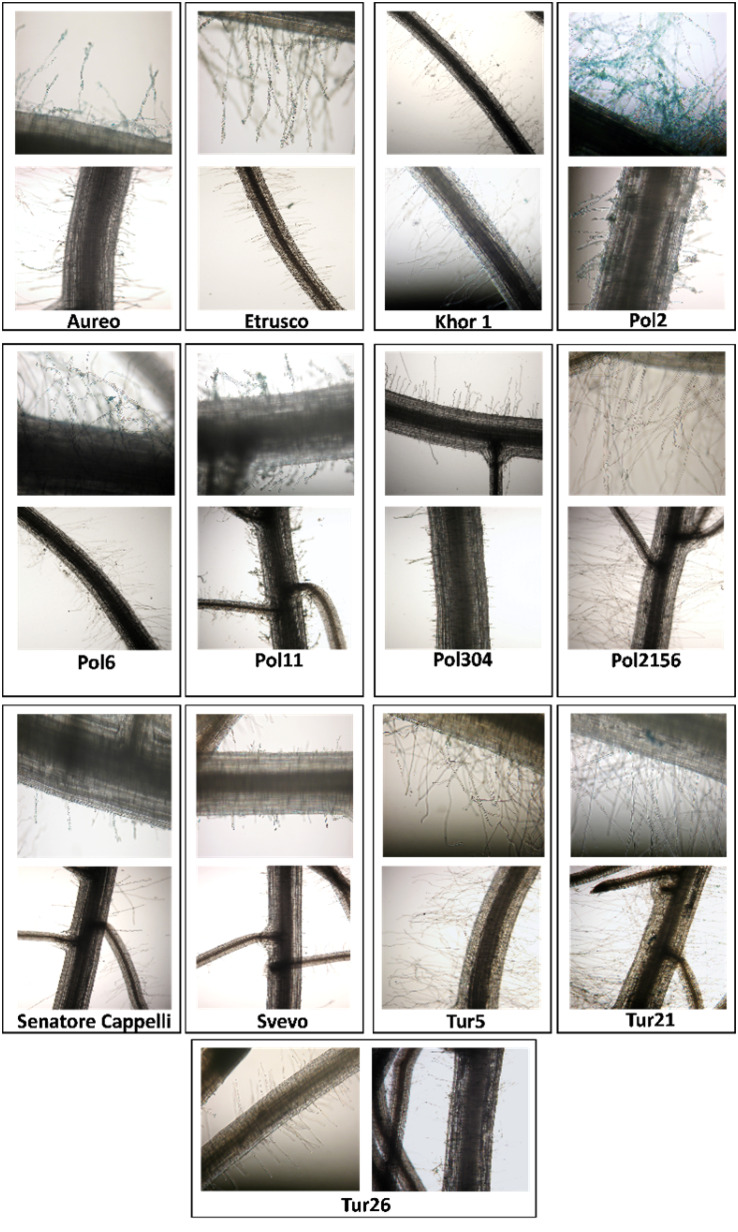
Visualization of iron deposits in roots of thirteen tetraploid wheat genotypes, using Perl’s staining technique.

The distribution and/or localization of ferric deposits in the root tissue of different durum wheat genotypes were evaluated using a very sensitive Fe-specific histochemical procedure. Obtained images showed that iron deposits, indicated by blue staining, were mainly localized at the level of the central zone of the primary root and the root hairs present in the same zone (**Figure 7**). Interestingly, the highest level of accumulation was observed for Pol2, as evidenced by the more intense blue staining than that obtained with the other genotypes (**Figure 7**).

## 4 Discussion

On the basis of the growing interest of consumers in wheats present before the Green Revolution, collectively named “ancient wheats,” parallel research is increasing to analyze them more in detail both from the nutritional and technological points of view (Cabas-Lühmann et al., 2023; Roumia et al., 2023).

In this paper, we have analyzed accessions of the *T. turgidum* group, including the subspecies *T. durum* or durum wheat, *T. polonicum* or Polish wheat, and *T. turanicum* or Khorasan wheat, grown in the same environment for 2 years, in terms of technological and nutrition-related aspects. Moreover, the same accessions have been hydroponically grown to test root morphology in order to possibly associate some of the characteristics that have been identified.

As regards the technological analyses, although protein and gluten contents were comparable among the accessions and in the 2 years and coherent with the average Italian cultivars (De Santis et al., 2017), the modern variety Svevo, as expected, had the best performance followed by Khor1. It is important to underline that Khor1 corresponds to the local field cultivation of KAMUT^®^ brand wheat; thus, it was not possible to use this name for locally produced kernels because they were obtained out of the official KAMUT^®^ food chain. KAMUT^®^ brand wheat has been selected also because of its better yield and technological performance; thus, it is expected that some of the qualitative parameters here measured are very good for Khor1 compared with that for other Khorasan or Polish wheats.

The test weight, a physical quality characteristic desirable because of its positive influence on market grade and price (Troccoli and Di Fonzo, 1999), showed a significant difference during the two crop years, with the first with significantly lower values probably due to three-fold more rainfall than in the second year. As reported in Kuzmanović et al. (2021), the 2019 season, at Nello Lupori experimental farm in Viterbo, was characterized by a high amount of rainfall and lower temperatures during the grain filling and the authors hypothesized that these conditions led to greater plant tiller number with less seed weight (meteorological data relative to the same location and years).

The yellow index was highly different among accessions, but not between the two growing seasons. This also is expected on the basis of the great heritability of this character (Colasuonno et al., 2019). In this case, also, Svevo and Khor1 showed the highest value.

The micro- and macro-nutrient amounts were measured because of their great nutritional importance (Khalid et al., 2023) and for their healthy beneficial effects such as antioxidant, anti-cancer, and anti-viral properties (Welch and Graham, 2004; Hansch and Mendel, 2009; Abbaspour et al., 2014). Furthermore, there is a common belief that ancient wheats contain a higher amount of these minerals (Shewry et al., 2016; Lovegrove et al., 2020; Shewry et al., 2020).

In our case, by considering the single accessions, the ANOVA analysis indicated that selenium variance was mostly explained by the genotype. In contrast, Lyons et al. (2005) reported that the amount of selenium in the kernels of wheat cultivars from Australia and Mexico is primarily determined by its availability and concentration in the soil. In fact, although higher concentrations of selenium, zinc, lithium, magnesium, and phosphorus were found in hulled wheat (*Triticum spelta* L. and *Triticum dicoccum* Schrank) grown together with modern bread wheats (Piergiovanni et al., 1997), several studies found no evidence for genetic variability among wheat cultivars for selenium accumulation in grain (Grela, 1996; Tveitnes et al., 1996; Zhao et al., 2009; Souza et al., 2014). However, a considerable genotypic variation and capacity in accumulating selenium, at least in root and shoot tissues, by four different tetraploid wheats, have been recently demonstrated (Coppa et al., 2023). In particular, the different selenate uptake ability was mainly explained by the differential expression of genes encoding the two high-affinity transporters (TdSultr1.1 and TdSultr1.3), which are involved in the primary uptake of sulfate (and selenate) from the growth medium (Coppa et al., 2023). In our work, by considering the different subspecies, the selenium concentration was lower in the Khorosan wheat in both growing seasons, but the differences were only partially explained by subspecies, year, and subspecies × year.

Our analyses highlighted that the accumulation of zinc among different accessions is mostly influenced by the genotype and interaction between genotype and year. This observation agrees with previous studies that reported a wider genetic variation for iron and zinc concentrations in diploid and tetraploid species such as *T. monococcum*, *T. turgidum* ssp. *dicoccoides*, *T. boeoticum*, *Aegilops tauschii*, *T. spelta*, *T. turgidum* spp *polonicum*, and some landraces (Cakmak, 2007; Velu et al., 2014).

The concentration of cadmium among different accessions is mostly influenced by the genotype and interaction between genotype and year, whereas, by considering the different subspecies, its concentration was significantly higher in durum and Khorasan wheat. This heavy metal, in recent years, has become one of the major and widespread soil pollutants due to intensive agricultural practices, unsustainable industrialization, and urbanization. Being highly toxic for plants, animals, and humans (Sarwar et al., 2010), there is growing interest in the selection of varieties that accumulate low cadmium levels. Cadmium accumulation in plants is determined by several genes that regulate mechanisms underlying its uptake, translocation, and accumulation (Tao and Lu, 2022). A class of metal transporters called Natural Resistance-Associated Macrophage Proteins (NRAMPs) was first reported in the model plant *Arabidopsis thaliana* involved in cadmium transport. Recently, three genes (*TpNramp3*, *TpNramp5*, and *TtNramp6*) encoding plasma membrane proteins involved in cadmium concentration and accumulation in the whole plant were found in *T. polonicum* and *T. durum*. Furthermore, Maccaferri et al. (2019) found that the modern durum wheats possessing the non functional copy of the *TdHMA3-B1A* gene accumulated higher amount of cadmium. Together, these results suggest that there are allelic variants in these genes in the different subspecies that regulate cadmium accumulation.

In general, by considering the different subspecies, the effect of the year among the three groups of subspecies was significant for all the minerals.

Finally, the results showed that the most interesting accessions were the following: Pol2, which accumulated the largest amount of zinc, magnesium, iron, and copper and had the lowest concentration of cadmium during the two seasons; Tur5, which showed the greatest accumulation of zinc, copper, and cadmium and the lowest accumulation of selenium; Senatore Cappelli, which accumulated a low amount of magnesium and copper but a high amount of cadmium; and Pol304, which showed high accumulation of iron and selenium. Interestingly, Pol2 showed the highest level of iron accumulation in root tissues, most likely suggestive of a significant apoplastic iron pool (Bienfait et al., 1985), but its iron concentration in seeds indicates a low relative efficiency of the “root-to-seed” iron transfer. Nutrient acquisition by roots is a key component of nutrient use efficiency (NutUE), but it is not the only one at least for iron. Indeed, it has been demonstrated that although the supply of iron from soil to the root apoplast should be adequate, the transport from the apoplast to the symplast is often impaired (Mengel, 1994); thus, even if a plant shows high concentrations of iron at root level, it can, however, show iron deficiency symptoms (Kosegarten and Koyro, 2001). High amounts of extraplasmatic iron have been detected in the roots of plants grown in nutrient solution (Bienfait et al., 1985), because, in roots, iron is mainly present as apoplastic pool, and this iron is basically adhering to the outer surface of the epidermis, probably in a particular form, and does not contribute significantly to the nutrition of the plant (Strasser et al., 1999). Furthermore, Grusak and Cakmak (2005) demonstrated that, even at high iron supply, the concentration of iron in shoot and seed could remain relatively low, likely due to limited phloem mobility. Therefore, the promising lines rich in micro- and macro-nutrients and poor in toxic elements such as cadmium identified in this study must be validated in multilocation trails. This study confirmed that a large variation in the concentrations of microelements is present among different accessions and is not solely intra-subspecies, because the environment and soil nutrient status are known to significantly affect these traits (Shewry et al., 2020).

The fibers (that include β-glucan and arabynoxylan) influence the quality of pasta and bakery products, due to their high capacity to bind the water present in the dough and to increase its viscosity. Furthermore, they have numerous beneficial physiological effects for humans, i.e., reduction of blood cholesterol, attenuation of blood glucose levels, and elevated prebiotic activity (Botticella et al., 2021). We found that, by considering the single accessions, the main contribution to the differences in WE-AX and β-glucan content was due to genotype, whereas TOT-AX content was mostly influenced by genotype-by-year interaction, in addition to by genotype. These results agree with the previous studies reported by Marion and Saulnier (2020). In general, Lovegrove et al. (2020), by analyzing 39 wheat cultivars dated between 1790 and 2012, showed that the environment affects the accumulation of health-related components, among which arabinoxylans, to a great extent. Instead, considering the subspecies, only for TOT-AX and β-glucan, there were significant effects, and, only for WE-AX and β-glucan, there were significant effects for the year. No significant effects were found for the interaction subspecies × year. In both growing season, durum wheats showed the highest content of β-glucan and TOT-AX, whereas there are no difference among the subspecies for the WE-AX content.

Root system plays a crucial role in plant fitness as roots are responsible for the efficient uptake of water and mineral nutrients. Therefore, root development and morphology were related to quality parameters (in terms of yield and technological and nutritional quality). In particular, the S/R is a measurement of the amount of shoot biomass (growth function) compared with the amount of root biomass (supportive function) and is generally maintained within a certain balance that is characteristic of the species. However, in this study, only the modern variety Svevo showed a well-balanced S/R, thus an optimal distribution of resources between the aboveground and belowground portions of the plant. This allocation should enable more efficient utilization of water and nutrients. On the other hand, the lowest values of all measured root morphological traits were observed in Svevo. Tur21 and Tur26 clustered together and showed the highest values of all morphological traits, as well as Pol2 and Pol6 clustered together and showed an increase in all roots morphological parameters compared with Svevo. Nutrient uptake in plants is, in fact, closely related to root morphology. In particular, the length and surface area of plant roots, as well as the number of root hairs, are directly related to their capacity to absorb nutrients. Therefore, although Pol2 had a lower root biomass, the particular morphology of the roots is more efficient for nutrient uptake, as evident from the greater accumulation of micro- and macro-nutrients.

In conclusion, the two accessions of Polish wheat Pol2 and Pol6 have a potential good technological quality. Moreover, Pol2 is rich in health- related micro- and macro-nutritients and poor in toxic ones Thus, these two accessions may be taken into consideration for the development of new varieties.

More information about dynamics of iron distribution in shoots and seeds is crucial to guide the exploitation of these findings to possibly improve the nutritional value of grains by increasing iron content. Thus, further experiments will explore the diversity of these wheats, with special interest in their relative efficiency of the “root-to-shoot” and “shoot-to-seed” iron transfer.

Finally, these results confirm that it is not possible to draw general conclusions about the difference between old and modern wheats, but rather a case-by-case approach should be taken into consideration, by including both the different genotypes and growing conditions.

## Data availability statement

The original contributions presented in the study are included in the article/**Supplementary Material**. Further inquiries can be directed to the corresponding authors.

## Author contributions

DL, FS, and SM: conceptualization; SP and MB: analysis of mineral and fiber content; AC: quality-related analysis, GQ and SA: root characteristics analyses, SP, MB, AC, GQ, SA, FS, and SM: writing the original draft; SP, MB, AC, GQ, SA, DL, FS, and SM: review and editing. All authors contributed to the article and approved the submitted version.
