# Corrigendum: Characterization of Triticum turgidum sspp. durum, turanicum and polonicum grown in Central Italy in relation to technological and nutritional aspects

**DOI:** 10.3389/fpls.2024.1379685

**Published:** 2024-03-06

**Authors:** Samuela Palombieri, Marco Bonarrigo, Alessandro Cammerata, Giulia Quagliata, Stefania Astolfi, Domenico Lafiandra, Francesco Sestili, Stefania Masci

**Affiliations:** ^1^ Department of Agriculture and Forest Science (DAFNE), University of Tuscia, Viterbo, Italy; ^2^ Council for Agricultural Research and Economics, Research Centre for Engineering and Agro-Food Processing, Rome, Italy

**Keywords:** *Triticum turgidum*, *Triticum durum*, *Triticum turanicum*, *Triticum polonicum*, technological quality, micronutrients, fiber, root traits

In the published article, there was an error in the **legend** for **Table 1** as published. There are mistakes regarding the use of KAMUT brand wheat name. Kamut International requested the change.

**Table 1:** Species, cultivars and accessions used in this paper.

Species	Original accession code or cultivar	New accession code
*T. turanicum*	PI624217	Tur5
*T. turanicum*	PI184543	Tur21
*T. turanicum*	PI576854	Tur26
*T. turanicum*	QK-77	Khor1*
*T. turanicum*	Etrusco	Etrusco
*T. polonicum*	PI191810	Pol2
*T. polonicum*	PI191808	Pol6
*T. polonicum*	CLTR5023	Pol11
*T. polonicum*	AS304	Pol304
*T. polonicum*	IC12196	Pol2156
*T. durum*	Aureo	–
*T. durum*	Svevo	–
*T. durum*	Senatore Cappelli	–

*Khor1 corresponds to kernels obtained by local field cultivation of KAMUT® brand wheat, purchased by Molino Bongiovanni s.r.l (Villanova Mondovì, CN, Italy). KAMUT® trademark is used to market and sell the QK-77 variety with certain quality guarantees. Because KAMUT® is a registered trademark of Kamut International Ltd. and Kamut Enterprises of Europe bv, this name could not be used for locally produced kernels because they were obtained out of the official KAMUT® food chain.

The corrected legend appears below.

“*Khor1 corresponds to kernels obtained by local field cultivation of KAMUT® brand wheat, purchased by Molino Bongiovanni s.r.l (Villanova Mondovì, CN, Italy). KAMUT® trademark is used to market and sell the QK-77 variety with certain quality guarantees. Because KAMUT® is a registered trademark of Kamut International Ltd. and Kamut Enterprises of Europe bv, this name could not be used for locally produced kernels because they were obtained out of the official KAMUT® food chain.”

In the published article, there was an error in **Table 1** as published. Kamut is not a cultivar neither an accession, thus Kamut has been replaced by the cultivar name that is QK-77.

The corrected **Table 1** and its caption appear below.

In the published article, there was an error. It is regarding the use of the name KAMUT.

A correction has been made to **Introduction**, Paragraph Number 3.

This sentence previously stated:

“The importance of the two subspecies has increased in the last decades, as a consequence of the introduction in the market of Kamut®, a specific accession of *T. turanicum* that has reached a high popularity because of its suggested positive effects on human health (Bordoni et al., 2017; Spisni et al., 2020). In the wake of Kamut®, any Khorasan wheat is acquiring importance because it is considered healthy (Geisslitz and Scherf, 2021), with some accessions sold under registered names and others simply described as such, sold as semolina or processed foods, with a wide market, especially in Western Countries where they can be found even in the large-scale distribution.”

The corrected sentence appears below:

“[The importance of the two subspecies has increased in the last decades, as a consequence of the introduction in the market of KAMUT® brand wheat, an ancient variety of grain (*Triticum turgidum* ssp. *turanicum*) that has reached a high popularity because of its suggested positive effects on human health (Bordoni et al., 2017; Spisni et al., 2020). In the wake of KAMUT® brand wheat, any Khorasan wheat is acquiring importance because it is considered healthy (Geisslitz and Scherf, 2021), with some accessions sold under registered names and others simply described as such, sold as semolina or processed foods, with a wide market, especially in Western Countries where they can be found even in the large-scale distribution.]”

In the published article, there was an error. It is regarding the use of the name KAMUT.

A correction has been made to **Discussion**, Paragraph Number 3.

This sentence previously stated:

“As regards the technological analyses, although protein and gluten contents were comparable among the accessions and in the 2 years and coherent with the average Italian cultivars (De Santis et al., 2017), the modern variety Svevo, as expected, had the best performance followed by Khor1. It is important to underline that Khor1 corresponds to the local field cultivation of Kamut®; thus, it was not possible to use this name for locally produced kernels because they were obtained out of the official Kamut® food chain. Kamut® has been selected also because of its better yield and technological performance; thus, it is expected that some of the qualitative parameters here measured are very good for Khor1 compared with that for other Khorasan or Polish wheats.”

The corrected sentence appears below:

“As regards the technological analyses, although protein and gluten contents were comparable among the accessions and in the 2 years and coherent with the average Italian cultivars (De Santis et al., 2017), the modern variety Svevo, as expected, had the best performance followed by Khor1. It is important to underline that Khor1 corresponds to the local field cultivation of KAMUT® brand wheat; thus, it was not possible to use this name for locally produced kernels because they were obtained out of the official KAMUT® food chain. KAMUT® brand wheat has been selected also because of its better yield and technological performance; thus, it is expected that some of the qualitative parameters here measured are very good for Khor1 compared with that for other Khorasan or Polish wheats.”

The authors apologize for these errors and state that this does not change the scientific conclusions of the article in any way. The original article has been updated.

