# Supplementary material for: Characterization of Triticum turgidum sspp. durum, turanicum, and polonicum grown in Central Italy in relation to technological and nutritional aspects
Source: Front Plant Sci. 2023 Dec 6;14:1269212. doi: 10.3389/fpls.2023.1269212 (PMC10731273; doi:10.3389/fpls.2023.1269212)
Supplement: Supplementary file 2 [file DataSheet_1.pdf]

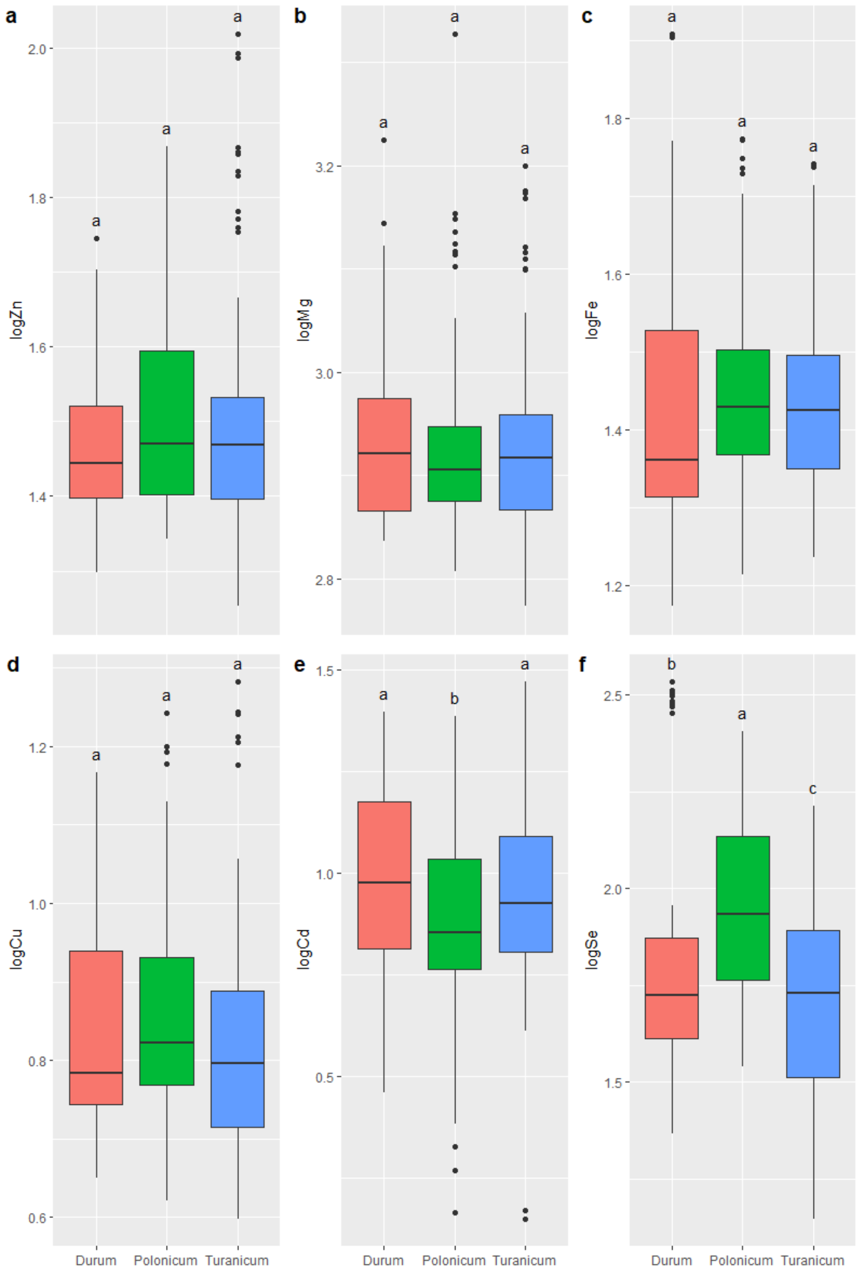

Figure S1 – Microelements content for subspecies. a) zinc, b) magnesium, c) iron, d) copper, e) cadmium, f) selenium. Boxplots with different letters are significantly different (ANOVA with Tukey's HSD, α = 0.05).

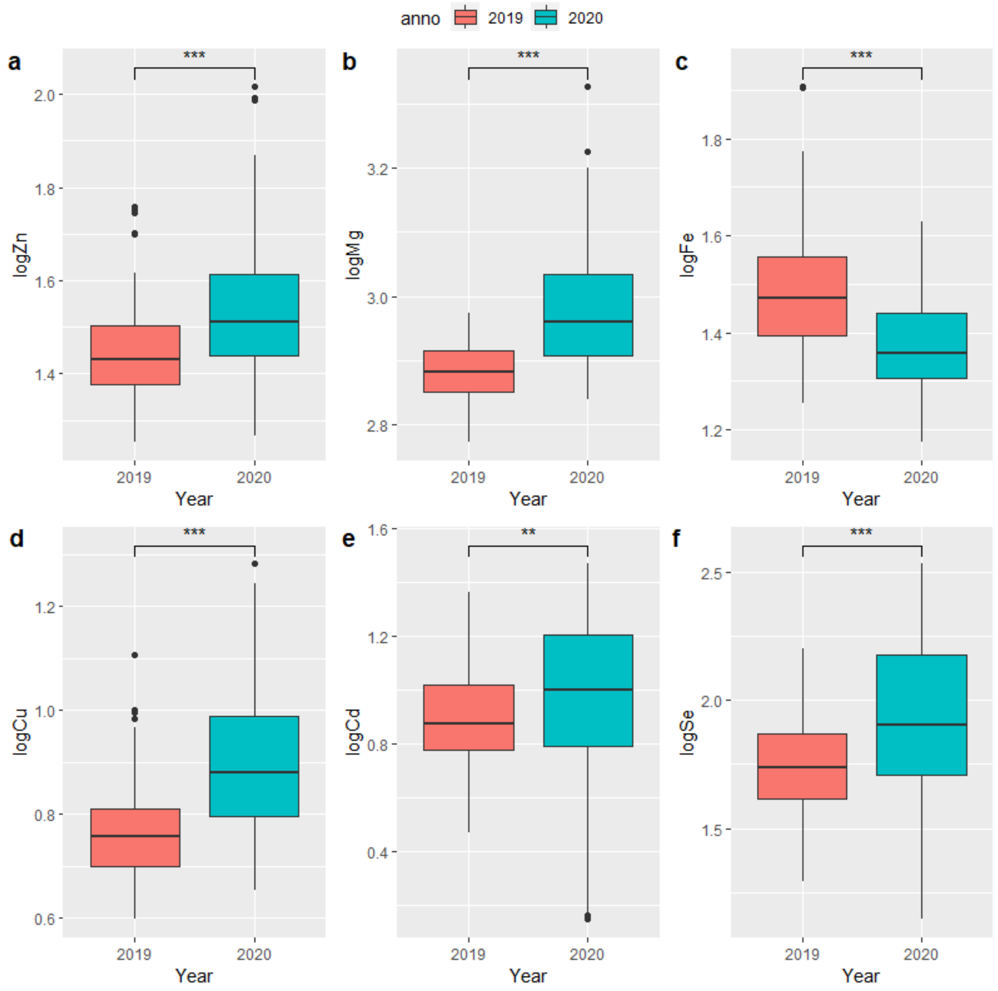

Figure S2 – Microelements content for year. a) zinc, b) magnesium, c) iron, d) copper, e) cadmium, f) selenium. Asterisk indicates significative differences between the years (Student t-test, α = 0.05). ** = p-value < 0.01; *** = p-value < 0.001.

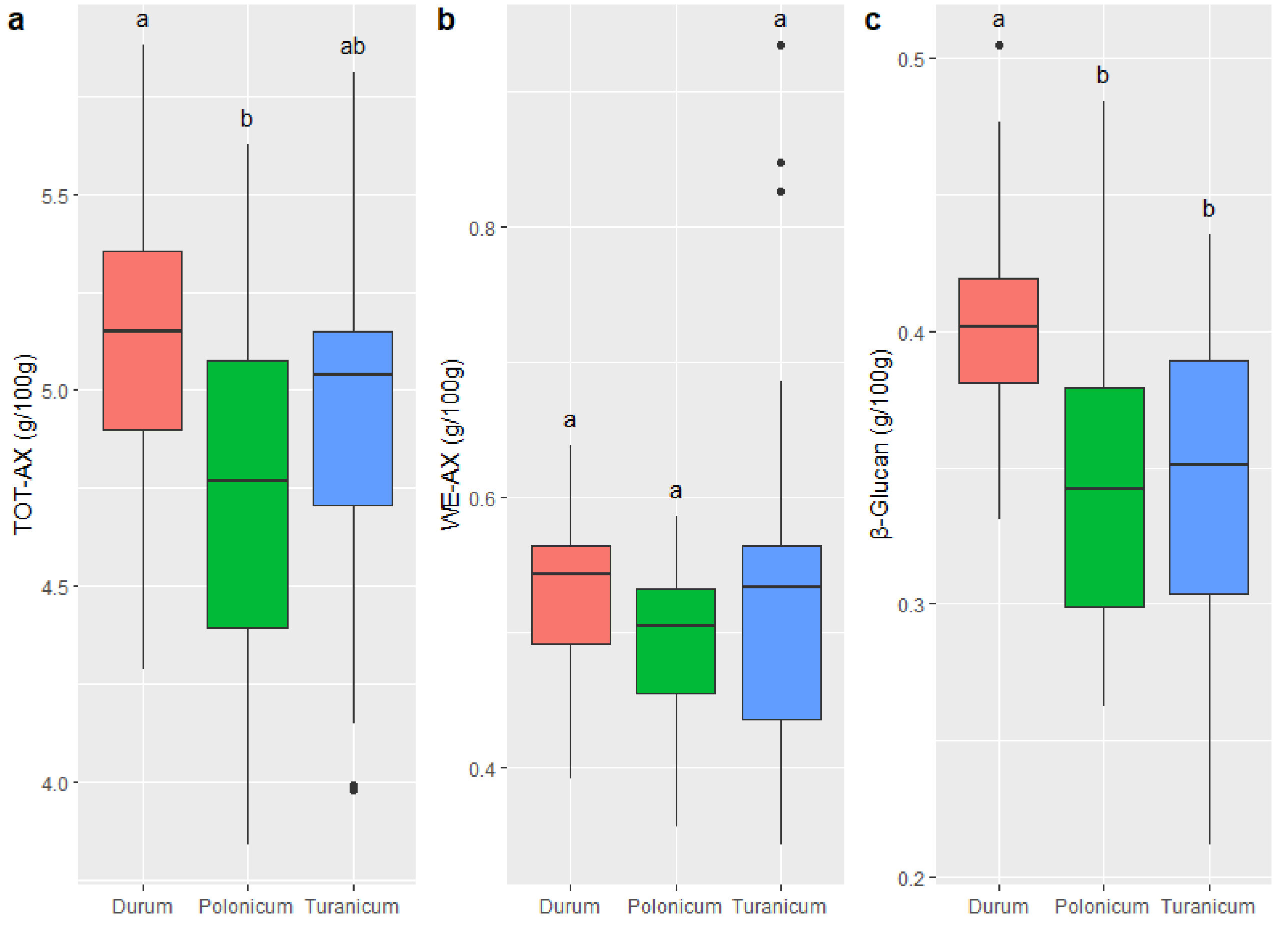

Figure S3 –Fiber content for subspecies a) total arabinoxylan, b) water-extractable arabinoxylan, c) β-glucan. Boxplots with different letters are significantly different (ANOVA with Tukey's HSD, α = 0.05).

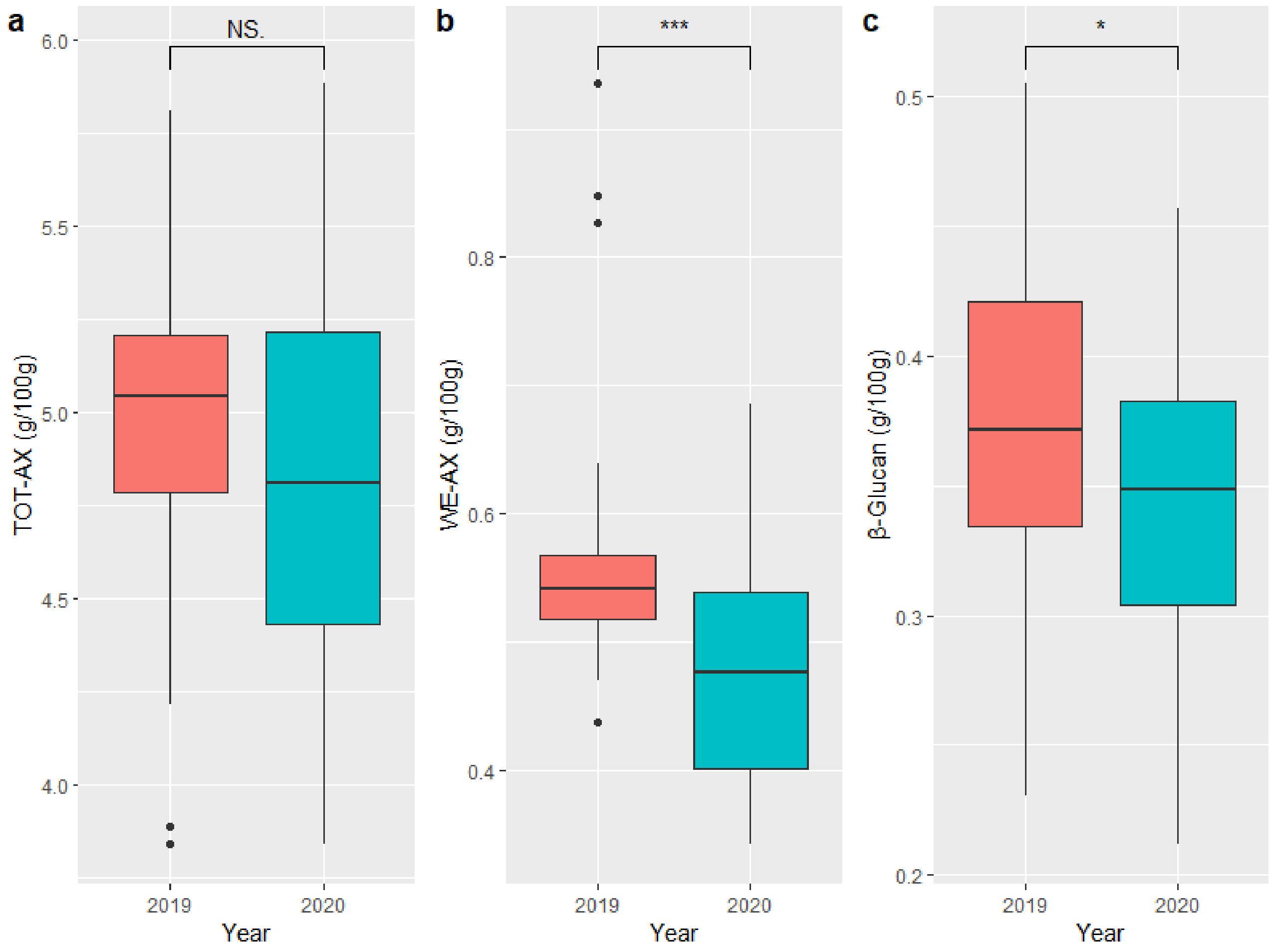

Figure S4 – Fiber content for year. a) total arabinoxylan, b) water-extractable arabinoxylan, c) β-glucan. Asterisk indicates significative differences between the years (Student t-test, α = 0.05). * = p-value < 0.05; *** = p-value < 0.001; NS. = p-value > 0.05.
